# Short-term crop rotation interactive effects on soil microbial communities and potato yield under field conditions

**DOI:** 10.3389/fmicb.2026.1807765

**Published:** 2026-04-29

**Authors:** Nyasha Esnath Chiuta, Kgabo Martha Pofu, Phatu William Mashela

**Affiliations:** Department of Plant Production, Soil Science and Agricultural Engineering, Faculty of Science and Agriculture, Green Biotechnologies Research Centre of Excellence, University of Limpopo, Sovenga, South Africa

**Keywords:** cropping system, enzyme activity, microbial diversity, potato, soil health

## Abstract

**Introduction:**

The inclusion of plant-parasitic nematode (*Meloidogyne* species) non-host crops in potato (*Solanum tuberosum* L.) -based cropping systems can sustainably manage the pest and reduce the application of greenhouse gas-emitting agro-chemicals. Nevertheless, there exists a research gap on how these cropping systems interact with soil microbiome.

**Methods:**

A 2-year field study was conducted at the University of Limpopo and the Agricultural Research Council to investigate the effects of potato monoculture, *Cucumis africanus*-potato, sorghum-potato and potato (Velum)-potato cropping sequences on soil health and tuber yield. The treatment crops, namely, sweet stem sorghum (*Sorghum bicolor*) cv. ′Ndendane-X1′, *C. africanus*, potato cv. ′Mondial G3′ and potato cv. ′Mondial G3′-velum (control), were assigned to 4 m^2^ subplots in Sequence 1, in a randomized complete block design, with six replicates. The successor potato crop was planted in Sequence 2. Crop arrangement in Sequences 3 and 4 was as in Sequences 1 and 2, respectively. Soil organic carbon content, enzyme activity, nematode and bacterial functional diversity data were collected from Sequences 1 to 4. Final potato tuber yield was recorded in Sequence 4.

**Results:**

Based on the results, nematode indices revealed disturbed agroecosystems dominated with bacterial decomposition pathways, which is a common occurrence in intensively managed soils. High soil organic carbon content, microbial diversity and enzyme activity were observed in sorghum-potato and *C. africanus*-potato compared to potato monoculture (with or without velum), at both locations. In addition, sorghum-potato improved soil structure over time based on the nematode faunal results, thereby depicting its ability to promote soil health compared to other cropping systems. Tuber yield mass significantly varied (*p* ≤ 0.05) among cropping sequences at both locations with potato (Velum)-potato and *C. africanus*-potato sequences recording higher yields than potato monoculture or sorghum-potato.

**Discussion:**

Crop diversification increased soil organic carbon content, microbial diversity and enzyme activity. Overall, sorghum-potato effectively improved soil health and soil structure over time, better than the other cropping systems.

## 1 Introduction

Smallholder potato (*Solanum tuberosum* L.) farmers underestimate the damage caused by plant parasitic nematodes (PPNs), confusing the pest symptoms with those of abiotic stress (Kantor et al., 2024). Globally, PPNs cause an annual economic yield loss of more than US$150 billion, across several crops (Ning et al., 2022), with root-knot (*Meloidogyne* species) nematodes (RKNs) contributing the largest proportion to this damage (Conceição, 2025). However, including nematode resistant crops in potato-based cropping systems can be a sustainable nematode management strategy (Chiuta et al., 2025), relative to the application of synthetic agrochemicals, which are detrimental to soil health.

Soil health is a complex biological property of soil which is influenced by many factors, including crop sequences (Collange et al., 2011). It is defined as the continued capacity of soil to function as a vital living system to sustain biological productivity and promote plant, animal and human health (Yang et al., 2020). Healthy soil is associated with extensive biodiversity, rapid nutrient mineralization and high resilience and resistance to deterioration (Collange et al., 2011). Soil biodiversity refers to the variety of living organisms occurring within the soil whereas, nutrient mineralization is the degradation of organic compounds into inorganic minerals by microorganisms (Collange et al., 2011). In general, soil with high resilience and resistance to deterioration is stable enough to withstand various environmental pressures (Collange et al., 2011).

The inclusion of different crops in a cropping system significantly impacts soil health, even over a short time frame (Town et al., 2022; Zhong et al., 2016). Regardless of crop rotation being an ancient practice, research focused on plant-microbial interactions has gained attention over the past decade. An increase in soil health (Larkin, 2024; Shi et al., 2026; Xie et al., 2025) and tuber yield (Khakbazan et al., 2023; Larkin, 2024; Shi et al., 2026; Xie et al., 2025) was observed in potato-based cropping systems. However, the effectiveness of each cropping system heavily depends on trial duration (short or long term), crop type, climatic and edaphic factors etc. The assessment of soil microbial populations through carbon source utilization profiles and enzyme activity in mineralization of nutrients provides information on how small changes in agricultural practices can influence soil health (Habig et al., 2018; Yang et al., 2020).

The enzymes ß-glucosidase, acid phosphatase and urease have been proposed as indexes of mineralization for carbon, phosphorus and nitrogen, respectively (Malobane et al., 2020). However, studies investigating the effect of crop sequences on soil health are limited. In real-world systems, the introduction of some agro-practices aimed at solving certain problems (e.g., pest or disease management) might create unforeseen challenges. For example, the introduction of fumigant nematicides resulted in a wide array of challenges, which included the deterioration of soil health (Meena et al., 2020). Therefore, understanding the impact of each cropping system on these microbial communities is important for the development of sustainable pest management practices that enhance soil health and crop productivity.

Nematode communities are effective bioindicators for soil health since they occur at every trophic level of the soil food web (Treonis et al., 2018) and readily respond to agricultural practices (Li et al., 2022). In most cases, soil nematode communities are analyzed through assessing the feeding habits, functional guilds and nematode indices (Bongers and Bongers, 1998). Feeding habits refer to the different diets of the nematodes within a soil food web (Yeates et al., 1993). For example, nematodes that feed on plants, bacteria, fungi, other invertebrates, or a combination of diets are known as herbivores, bacterivores, fungivores, predators and omnivores, respectively (Pinto et al., 2024; Yeates et al., 1993). Functional guilds are groups of nematode species that have similar feeding habits within an ecosystem (Bongers and Bongers, 1998).

Nematode indices such as enrichment index (EI), structural index (SI), channel index (CI), plant parasitic index (PPI) and maturity index (MI) provide information about the enrichment, structure and maturity of the agroecosystem (DuPreez et al., 2022). These indices are calculated using the different trophic groups and coloniser-persister (c-p) values. The enrichment and structural indices explain the availability of nutrients and the complexity of the food web within an environment, respectively. From these two indices the faunal profile of the soil can be determined. The CI is used to determine the prevailing decomposition pathway in the ecosystem which can be bacteria or fungi-dominated (Ikoyi et al., 2023). The PPI gives the proportion of herbivorous nematodes occurring within a sample.

Maturity index is a measure of soil stability or disturbance whereby high MI values represent undisturbed, mature or pristine environments predominated with persister (K-strategist) nematodes and a few colonisers (r-strategist) nematodes and vice versa (Bongers and Bongers, 1998). Essentially, r-strategists are small nematodes which are highly responsive to nutrient enrichment, very tolerant to environmental disturbances, have a short generation time and produce many small eggs (Bongers and Bongers, 1998; DuPreez et al., 2022). Contrariwise, K-strategists are large nematodes that hardly respond to changes in the environment, have long generation time and females that reproduce fewer eggs (DuPreez et al., 2022). These indices, together with general ecological indices such as Shannon-Weaver and Simpson’s index, have been used to determine diversity and evenness of species within the nematode assemblages, respectively (Ikoyi et al., 2023). Nematode diversity refers to the variety of species that occur within an ecosystem, whereas evenness is the measure of the species’ relative abundance (Ikoyi et al., 2023).

This study investigated the impact of including pre-and-post-infectional nematode resistance crops when managing RKN in potato-based cropping sequence on tuber yield and soil health. Ideally, a good nematode management strategy should enhance crop yield and soil health. It was hypothesized that crop yield and soil health would improve with increased crop diversity. A better understanding of how different cropping sequences affect soil health can help farmers in making informed decisions before adopting new agricultural practices.

## 2 Materials and methods

### 2.1 Study area

The experiment was established in 2017 and conducted for two years at the University of Limpopo (UL), South Africa (23°53′10″S, 29°44′15″E) and at the Agricultural Research Council -Vegetable, Industrial and Medicinal Plants (ARC-VIMP), Roodeplaat, South Africa (25.61′44″S, 28.35′45″E), under field conditions, in soils that were predominantly infested with RKNs. The UL and ARC-VIMP sites fall under the semi-arid climatic region, with an average annual rainfall of 500 and 650 mm, respectively. A detailed description of the trial sites and history is given by Chiuta et al. (2025).

### 2.2 Experimental material

*Cucumis africanus* is a post-infectional nematode resistant crop which is indigenous to Limpopo Province, South Africa. Sweet stem sorghum [*Sorghum bicolor* (L.) Moensch] cv. ‘Ndendane-X1’ obtained from UL is a pre-infectional nematode-resistant crop. Seeds from *C. africanus* fruit and sorghum were used to produce seedlings under greenhouse conditions for use in this study. Potato cv. ‘Mondial G3’ seeds were sourced from commercial farmers selling seed potatoes.

### 2.3 Experimental design and management

Four cropping systems, namely, (i) potato monocropping, (ii) sorghum-potato rotation, (iii) *C. africanus*-potato rotation and (iv) potato (Velum)-potato were assessed in this study. Treatments were arranged in a randomized complete block design and replicated six times. A detailed experimental design is explained by Chiuta et al. (2025). Briefly, the treatment crops were randomly assigned to 2 m × 2 m sub-plots in a 11 m × 2 m block to establish Sequence 1 of the cropping systems. The successor potato crop was planted on all plots in Sequence 2. The treatment randomization established in Sequence 1 was maintained in Sequence 3 but Sequence 4 arrangement was like in Sequence 2.

The recommended commercial farming methods for potato [Department of Agriculture, Forestry and Fisheries (DAFF), 2010a] sweet stem sorghum [Department of Agriculture, Forestry and Fisheries (DAFF), 2010b] and *C. africanus* (Pofu, 2012) were carefully followed. Velum Prime nematicide was applied at planting in the furrow of potato (Velum) plots at the rate of 500 mL ha^−1^ in Sequence 1 and 3 only. Insect pests and diseases were managed as recommended for potato in commercial farming systems in South Africa (DAFF, 2010a), with other crops receiving similar pesticidal applications. Whiteflies (*Trialeurodes vaporariorum* Westwood) were controlled using Whitefly Insecticide (Pyriproxyfen, 50 g/L) from Efekto applied at 5 mL/ 5 L water. Preventative sprays against early blight and late blight were done once throughout the growing period using Tenazole 250 EW at 75 mL/100 L water and Mycoguard 720 SC at 1 L/ 500 L water/ ha, respectively. Copper-Flow-Plus (bactericide and fungicide) was applied once at 50 mL/ 10 L water to manage late blight that appeared to persist on potato. Weeds were manually removed by hand pulling and hoeing. The soil at UL is predominantly Hutton sandy loam (65% sand, 16% clay, 19% silt) with ECe 0.15 dS/m and 7.99 pH. At ARC-VIMP soil is sandy loam (8% clay, 60% sand, 32% silt), with EC_e_ 0.24 dS/m and 8.11 pH.

### 2.4 Data collection

Approximately 250 g soil samples were collected from the rhizosphere (20 cm) of each plant in each sub-plot at termination of each sequence, 56 days after trial establishment. The soil samples from the sub-plots of the same treatment were composited and mixed thoroughly using a 120 L Concrete Mixer (Turner Morris, Pretoria). The soil was sieved through a 2 mm mesh sieve to remove plant debris before storing samples in the cold room set at 4 °C prior to further analysis.

#### 2.4.1 Yield

Potato tubers were harvested from every plot. Tuber yield mass was measured using a CBK 8H Bench Check Weighing Scale balance (Adam Equipment, Oxford, UK) after terminating the last cropping sequence (Sequence 4).

#### 2.4.2 Soil organic carbon content

Approximately 100 g of soil subsample was air-dried for three days and ground using a pistil and a motor. The ground soil was passed through a 2–1 mm nested sieve to homogenize the soil. Soil organic carbon was quantified from a 1 g soil subsample using the Walkley-Black method (Walkley and Black, 1934). The amount of organic carbon in the soil sample was determined using the following equation


$$\text{Organic carbon}\%\text{in soil}=[\frac{(\mathrm{VB}-\mathrm{VS})\mathrm{ml}\times 0.003\times 100}{2\times M}]\times 1.3$$

Where, VB = blank titration reading.

VS = volume at endpoint of the sample reading.

M = mass of soil sample used.

#### 2.4.3 Bacterial functional diversity

The population and diversity of bacteria in the soil were determined using the Biolog EcoPlatesTM (Biolog® Inc., Hayward, USA). Briefly, a 10 g sieved soil sub-sample of each treatment crop was placed in a 200 cm^3^ beaker and diluted with 90 mL sterile distilled water. Portions of the samples were inoculated into Biolog EcoPlatesTM (Biolog® Inc., Hayward, USA) containing 31 carbon sources and a control well (water only), in triplicate. A multichannel pipette was used to dispense 0.15 mL aliquots into each EcoPlate well. The plates were then incubated in the dark at 28 °C. The rate of carbon source utilization by the microbial populations caused color changes from colorless to purple, which was measured using a Microplate reader (BioBase Biodustry, Shandong, China) set at 590 nm wavelength.

The functional diversity of the soil microbial populations was determined using the amount and equitability of carbon substrates metabolized in the Biolog EcoplatesTM as indicators of diversity (Shannon Weaver diversity index) and abundance (Evenness index), respectively (Habig et al., 2018). The Shannon-Weaver calculated as H′ = − *Σ* [pi In (pi)], where pi = ai/Σa, which is the proportional turbidity recorded in the i th well, ai = turbidity of the i th well and Σa = total turbidity of all sample wells (Lahav and Steinberger, 2013). The Evenness index (E) was calculated as E = H′/ In S, where H′ is the calculated Shannon Weaver diversity index, In is the natural logarithm of S is the number of species.

#### 2.4.4 Soil enzyme activity

ß-Glucosidase and acid phosphatase activities were analyzed from 1 g soil sub-samples using the Eivazi and Tabatabai (1988) and Eivazi and Tabatabai (1977) protocols, respectively. Glucosidase and acid phosphatase activities were calculated by determining the release of p-nitrophenyl using a DR3900 spectrophotometer (HACH, Randburg, South Africa) at 410 nm wavelength against a standard calibration curve produced by analyzing different concentrations of p-nitrophenol. The Kandeler and Gerber (1988) method was used to determine the activity of urease from approximately 5 g of soil sub-sample. Urease activity was determined by measuring the release of ammonia from the solution using a spectrophotometer set at 690 nm wavelengths. Results of urea were calculated with reference to a standard calibration curve obtained from analyzing different concentrations of urea (Habig et al., 2018).

#### 2.4.5 Nematode functional diversity

Nematodes were extracted from 250 g soil, sub-sampled using the sugar centrifugal flotation method (Marais et al., 2017). Nematode identification and enumeration were conducted by a nematologist at the Agricultural Research Council - Plant Health and Protection (ARC-PHP).

### 2.5 Data analysis

Tuber yield data were normally distributed based on the Shapiro–Wilk test. As such, data were exposed to analysis of variance (ANOVA) using SPSS version 17.0. Treatment means were compared at a probability level of 5% using Fisher’s Least Significant Difference test. The soil RKN population density data from Sequence 4 were subjected to a nonparametric Friedman test using the XLSTAT 2019 software, considering data did not meet all the assumptions for a parametric test. The treatment means were compared at the probability level of 5% using the Nemenyi test (Nemenyi, 1963; Pohlert, 2014).

Soil health data from each location were analyzed separately. Soil organic carbon, bacterial functional diversity and soil enzyme activity data were subjected to principal component analysis using XLSTAT 2019 software. Nematode community data were analyzed using the Ninja program (Sieriebriennikov et al., 2014). Each nematode taxa were assigned to different trophic groups and appropriate c-*p* values were assigned to each nematode genus (Bongers and Bongers, 1998). The c-p values were used to calculate MI, PPI, CI, EI, and SI. These indices were calculated using the following formulas,

1. Maturity index (MI) = Ʃ ((vi × fi)/n) where vi and fi are the c-p value and the frequency value of the taxon i in a sample, respectively and n is the total number of individuals present in a sample (Bongers, 1999).
2. Plant-parasitic index (PPI) = Ʃ ((vi × fi)/n), where vi is the cp-value of a family, fi is the frequency of the family in a sample and n is the total number of individuals present in a sample (Bongers, 1999).
3. Shannon-Weaver diversity index (H′) = − *Σ* [Pi In (Pi)], where Pi is the proportion of the ni in the nematode community n (Briar et al., 2012).
4. Channel index (CI) = 100(keFu2/(keBa1 + keFu2)), where keFu2 is the enrichment weightings of fungivores with a cp-2 value (Fu2), having a coefficient of 0.8, whereas keBa1 is the enrichment weightings of the bacterivores with a cp-1 value (Ba1), having a coefficient of 3.2 (Ferris et al., 2011).
5. Enrichment index (EI) = 100(e/(e + b)), where e = Ʃ kene, where ke and ne represent the structure of indicator weights and abundance of nematodes in those guilds, respectively, and b is calculated as Ʃkbnb, where kb and nb are the weighted constant of the guild and the number of nematodes in that guild, respectively (Ferris et al., 2011).
6. Structure index (SI) = 100(s/(s + b)), where s = Ʃ ksns, where ks and ns represent the structure of indicator weights and abundance of nematodes in those guilds, and b is calculated as described above (Ferris et al., 2011).

Enrichment and structure indices were used to compute the faunal profile of the samples collected per sequence using Microsoft Excel 2010.

## 3 Results

### 3.1 Effect of different cropping systems on final potato tuber yield

The results from ARC-VIMP showed that the final RKN soil population density in potato monoculture and sorghum-potato plots was similar and significantly (*p* ≤ 0.05) higher than that observed in potato (Velum)-potato plots (Table 1). However, the final RKN soil population density of *C. africanus*-potato plots was not significantly different (p ≤ 0.05) from the highest or the lowest recorded values (Table 1). On the other hand, Potato-(Velum)-potato and *C. africanus*-potato had significantly (p ≤ 0.05) high tuber yield mass relative to that of potato monoculture and sorghum-potato (Table 1).

**Table 1:** Final root-knot (*Meloidogyne* species) nematodes population densities per 250 g of soil collected from potato monoculture (P–P), sweet stem sorghum-potato (SSS–P), *Cucumis africanus*-potato (C–P) and potato(Velum)-potato (PV–P) cropping sequences and tuber yield recorded at the Agricultural Research Council-Vegetable, Industrial and Medicinal Plants (ARC-VIMP) and University of Limpopo.

Cropping sequence	ARC-VIMP		University of Limpopo	
	Final nematodes	Tuber yield (g/plant)	Final nematodes	Tuber yield (g/plant)
P–P	910.00^a^ ± 54.10	270.42^b^ ± 19.62	2646.67^a^ ± 186.95	190.26^b^ ± 12.30
SSS–P	713.33^a^ ± 69.02	293.09^ab^ ± 17.63	1390.00^ab^ ± 94.06	198.11^b^ ± 11.67
C–P	450.00^ab^ ± 21.13	313.09^a^ ± 10.59	550.00^b^ ± 61.05	232.63^a^ ± 13.80
PV–P	173.33^b^ ± 32.93	321.81^a^ ± 16.93	643.33^b^ ± 61.84	238.22^a^ ± 16.41

Means ± standard errors sharing the same lowercase letter within cropping sequences (column) were not significantly different (*p* ≤ 0.05) within a sequence according to Nemenyi’s post-hoc test and Fischer’s Least Significant Difference test for soil nematode population density and tuber yield mass, respectively.

At the UL location, the final RKN soil population density in potato (Velum)-potato and *C. africanus*-potato plots were similar and significantly (*p* ≤ 0.05) lower than that recorded under potato monoculture(Table 1). However, final RKN soil population density in sorghum-potato plots was not significantly different (*p* ≤ 0.05) from the highest or the lowest values recorded (Table 1). The tuber yield mass from potato (Velum)-potato and *C. africanus*-potato were significantly higher than that recorded under sorghum-potato and potato monoculture (Table 1).

### 3.2 Soil organic carbon, bacterial functional diversity and enzyme activity

**Figure 1:**
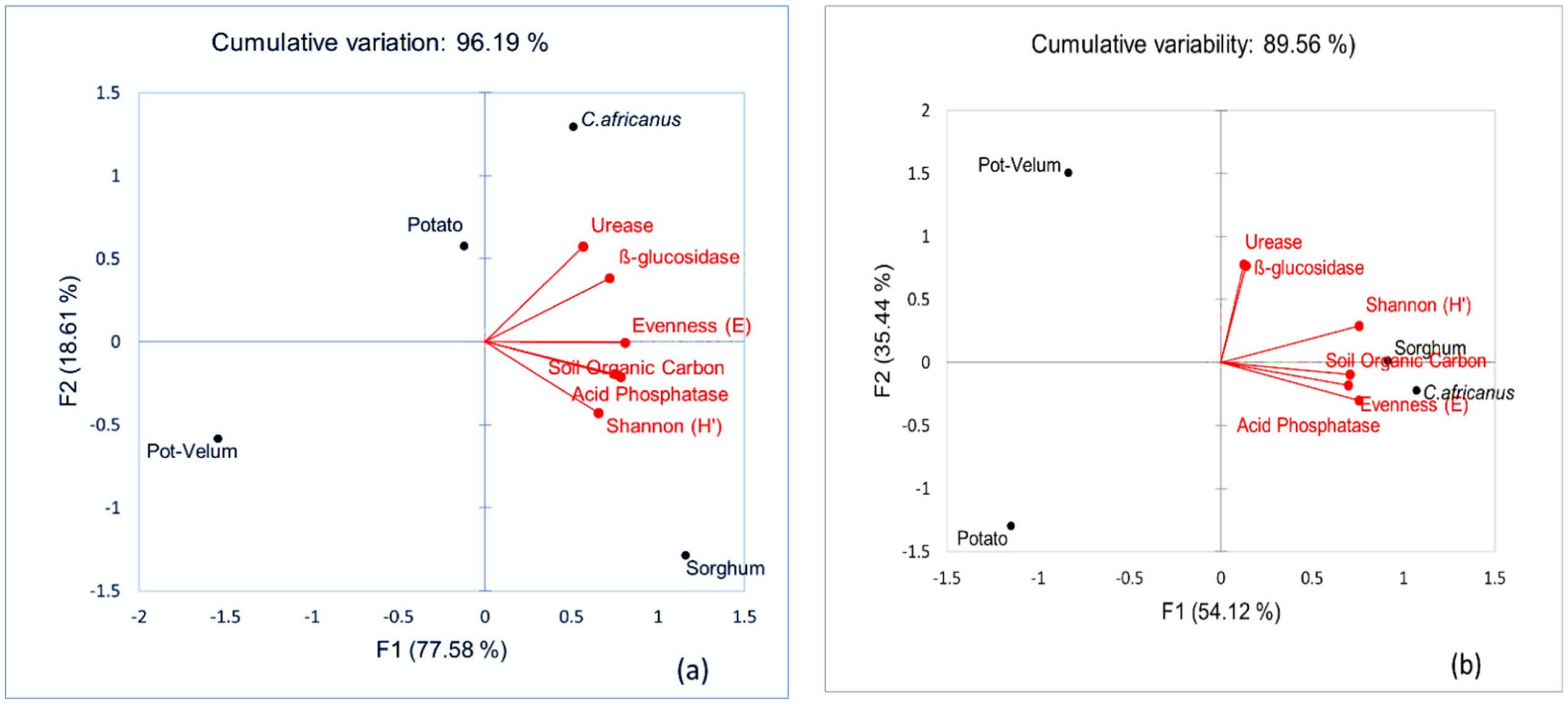
Biplot showing average soil organic carbon, microbial diversity, and enzyme activity occurring in potato monoculture (potato), sweet stem sorghum-potato (sorghum), *Cucumis africanus*-potato (*C. africanus*), and potato (Velum)-potato (Pot–Velum) cropping sequences at the University of Limpopo **(a)** and Agricultural Research Council-Vegetable, Industrial and Medicinal Plants **(b)**.

The average effect of each cropping system on soil organic carbon, bacterial functional diversity and enzyme activity at UL and ARC-VIMP was exhibited in Figures 1a, b, respectively. Principal components with an eigenvalue greater than one (Supplementary Table S1) were retained at both locations.

At UL, the total variation of the data sets in Figure 1a was explained by two components, PC 1 (77.58%) and PC 2 (18.61%), which had a cumulative variability of 96.19%. Factors such as ß-glucosidase enzyme activity, acid phosphatase enzyme activity, Shannon diversity, evenness index and soil organic carbon were strongly associated and received the highest loadings in PC1 (Supplementary Table S2). However, urease activity recorded the highest factor loading in PC2 and was highly correlated to ß-glucosidase enzyme activity (Supplementary Table S2). The different cropping systems were divided using PC 1. Based on the results, *C. africanus*-potato cropping sequence promoted urease and ß-glucosidase enzyme activity, whereas soil organic carbon, acid phosphatase enzyme activity and Shannon diversity were promoted by sorghum-potato cropping sequence (Figure 1a).

At ARC-VIMP two components, PC 1 (54.12%) and PC 2 (35.44%), with a cumulative variability of 89.56%, explained the total variation of the data sets (Figure 1b). The first principal component was strongly influenced by all the measured variables except urease and ß-glucosidase enzyme activity which strongly influenced PC2 based on the factor loadings (Supplementary Table S2). *Cucumis africanus*-potato cropping sequence promoted soil organic carbon, acid phosphatase enzyme activity and evenness index whereas Shannon diversity was strongly promoted by sorghum-potato cropping sequence (Figure 1b). In contrast, potato (Velum)-potato and potato monoculture were negatively correlated with all the measured variables (Figure 1b).

### 3.3 Nematode communities as an indicator for soil health

A total of 25 nematode genera with a c-p class ranging between 1 and 5 were identified from the soil samples obtained from S1 to S4 (Table 2). Generally, 48, 32, 12, 4 and 4% of the nematode species listed below (Table 2) were herbivorous, bacterivores, fungivores, predacious and omnivorous. *Tylenchorhynchus*, *Helicotylenchus*, *Scutellonema*, *Meloidogyne*, *Panagrolaimus* and *Aphelenchus* were the most abundant nematode species found across the sequences. In contrast, *Telotylenchus*, *Zeldia Elaphonema*, *Aphelenchoides*, *Eudorylaimus* and *Aporcelaimus* were the least represented nematode species.

**Table 2:** Functional diversity of nematodes found in four cropping sequences at the University of Limpopo (UL) and at the Agricultural Research Council-Vegetable, Industrial and Medicinal Plants (ARC-VIMP).

Genera	X	Y	Z	SEQ 1	SEQ 2	SEQ 3	SEQ 4
*Criconema*	0	3	HE	+		+	+
*Criconemoides*	0	3	HE	+	+		
*Nanidorus*	0	4	HE		+		
*Tylenchorhynchus*	0	3	HE	+	+	+	+
*Xiphinema*	0	5	HE			+	
*Paratylenchus*	0	2	HE			+	
*Paratrichodorus*	0	4	HE			+	
*Helicotylenchus*	0	3	HSE	+	+	+	+
*Telotylenchus*	0	2	HSE				
*Rotylenchus*	0	3	HSE	+	+	+	
*Scutellonema*	0	3	HSE	+	+	+	+
*Meloidogyne*	0	3	HS	+	+	+	
*Mesorhabditis*	1	0	Ba	+	+	+	
*Panagrolaimus*	1	0	Ba	+	+	+	+
*Acrobeles*	2	0	Ba	+	+		
*Acrobeloides*	2	0	Ba	+	+		
*Eucephalobus*	2	0	Ba		+	+	+
*Zeldia*	2	0	Ba	+			
*Elaphonema*	3	0	Ba				
*Cephalobus*	2	0	Ba		+	+	+
*Aphelenchoides*	2	0	Fu		+		
*Aphelenchus*	2	0	Fu	+	+	+	+
*Ditylenchus*	2	0	Fu	+		+	
*Eudorylaimus*	4	0	Pr	+			
*Aporcelaimus*	5	0	Om				+

x = coloniser-persister class, y = plant-parasitic class, z = Feeding type. HE = herbivores-endoparasites, HSE = herbivores - semi-endoparasites, HS = herbivores - sedentary, Ba = bacterivores, Fu = fungivores, Pr = predators, Om = Omnivores and SEQ = sequence.

#### 3.3.1 Feeding type composition of nematode assemblage

**Figure 2:**
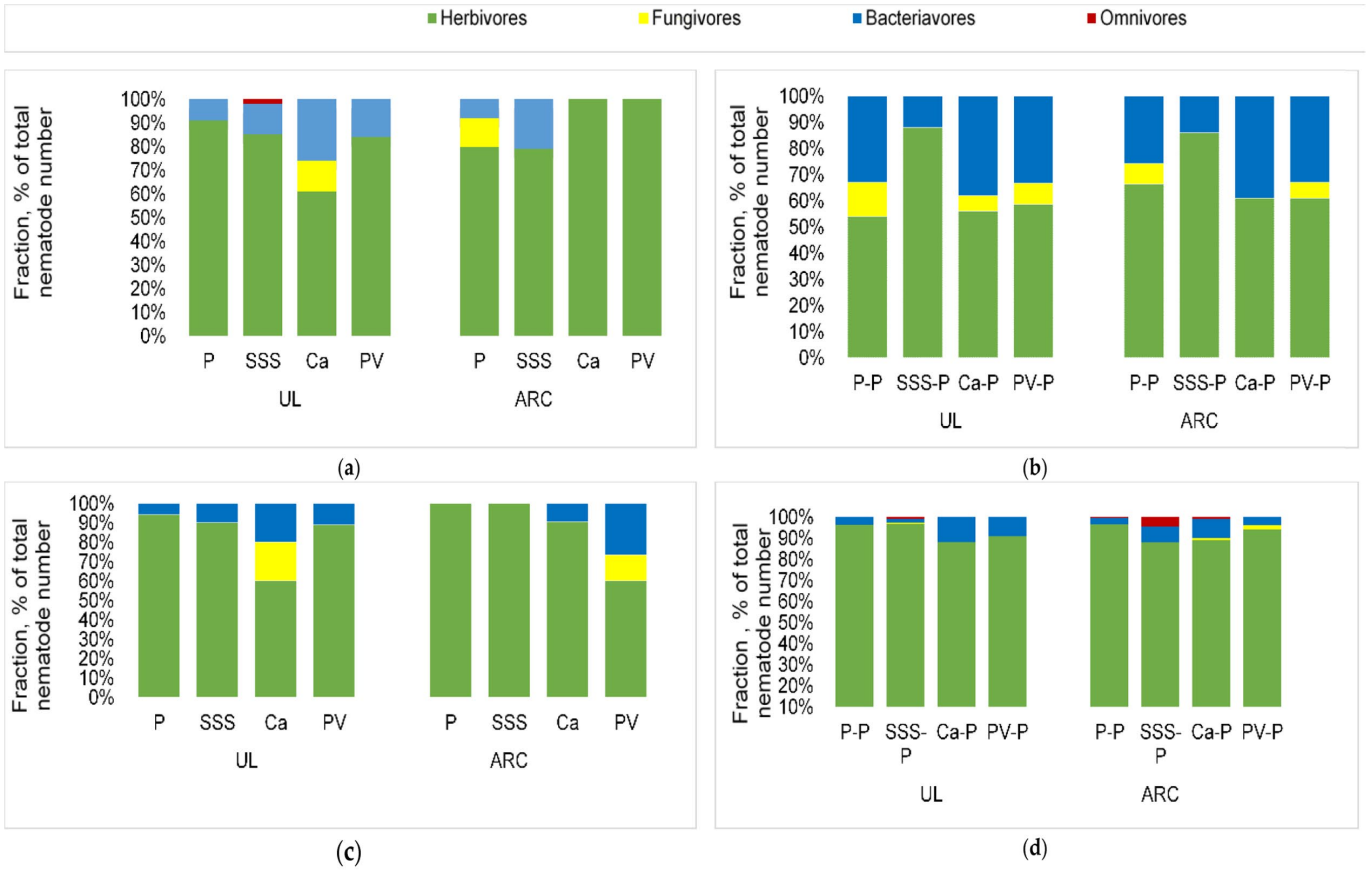
Feeding type composition of nematode assemblage of different cropping systems in sequence 1 **(a)**, sequence 2 **(b)**, sequence 3 **(c)**, and sequence 4 **(d)** at University of Limpopo (UL) and at the Agricultural Research Council (ARC), where P = potato, SSS = sweet stem sorghum, Ca = *Cucumis africanus*, PV = potato (Velum)-potato.

Herbivores were the most dominant nematodes in soil samples collected from all sequences at UL and ARC-VIMP. These were closely followed by bacterivores and fungivores (Figure 2). However, fungivores dominated all plots in Sequences 2 and 4, when sole potato cultivation was practiced, compared to when treatment crops were cultivated (Sequences 1 and 3).

Omnivores were the least dominating nematodes which were found in Sequence 1 and 4 of the sorghum-potato cropping system and in Sequence 4 of potato monoculture and *C. africanus*-potato plots at ARC. However, no omnivores were recorded in potato (Velum)-potato plots at both fields throughout the study (Figure 2).

#### 3.3.2 Nematode community indices

Maturity index of most cropping systems at UL and ARC-VIMP was low (< 2) throughout the study (Table 3). However, sorghum-potato recorded moderate MI values in Sequence 4 at UL and ARC-VIMP. Plant parasitic index was similar in all plots throughout the study except in Sequence 3 when *C. africanus* recorded the lowest PPI value at UL (Table 3). Generally, low CI values dominated most of the treatment plots at both UL and ARC-VIMP. However, extremely high CI was recorded on potato monoculture (Sequences 1 and 2), *C. africanus*-potato and potato (Velum)-potato (Sequence 4) at ARC, whereas at UL, high C. I value was observed on sorghum-potato in Sequence 4. The values for Shannon diversity index were very low (≤ 2). Therefore, low nematode species richness was observed on all the plots throughout the study.

**Table 3:** Maturity index (MI), plant-parasitic index (PPI), channel index (CI) and Shannon diversity (H′) of different cropping sequences at the University of Limpopo (UL) and at Agricultural Research Council-Vegetable, Industrial and Medicinal Plants (ARC-VIMP).

Sequence	Location	Treatment	MI	PPI	CI	H′
1	UL	P	2.00	3.00	0.00	0.93
		SSS	1.78	3.00	0.00	1.61
		CA	1.67	3.00	20	1.83
		PV	1.00	3.00	100	0.84
	ARC-VIMP	P	2.00	3.00	0.00	0.99
		SSS	1.50	3.00	0.00	1.41
		CA	0.00	3.00	0.00	1.04
		PV	0.00	3.00	0.00	0.00
2	UL	P → P	1.86	3.12	33.30	2.00
		SSS → P	1.00	3.00	0.00	1.28
		CA → P	1.43	3.00	5.88	1.84
		PV → P	1.70	3.00	14.29	1.51
	ARC-VIMP	P → P	2.00	3.00	100.00	1.44
		SSS → P	1.00	3.16	0.00	1.45
		CA → P	1.59	3.00	0.00	1.42
		PV → P	1.65	3.00	9.68	1.91
3	UL	P → P → P	1.00	3.00	0.00	1.00
		SSS → P → SSS	1.00	3.06	0.00	1.49
		CA → P → CA	1.50	2.67	20.00	1.36
		PV → P → PV	2.00	3.50	0.00	1.68
	ARC-VIMP	P → P → P	0.00	3.00	0.00	0.00
		SSS → P → SSS	0.00	3.00	0.00	1.05
		CA → P → CA	2.00	3.00	0.00	1.61
		PV → P → PV	1.67	3.11	20.00	1.88
4	UL	P → P → P → P	2.00	3.00	0.00	0.49
		SSS → P → SSS → P	2.86	3.00	100.00	0.29
		CA → P → CA → P	2.00	3.00	0.00	0.47
		PV → P → PV → P	2.00	3.00	0.00	0.30
	ARC-VIMP	P → P → P → P	2.38	3.00	0.00	0.30
		SSS → P → SSS → P	3.06	3.00	0.00	1.05
		CA → P → CA → P	2.25	3.00	100.00	0.66
		PV → P → PV → P	2.00	3.00	100.00	0.77

Where P = potato, SSS = sweet stem sorghum, Ca = Cucumis africanus, PV = potato-Velum cropping sequences.

#### 3.3.3 Faunal profile

**Figure 3:**
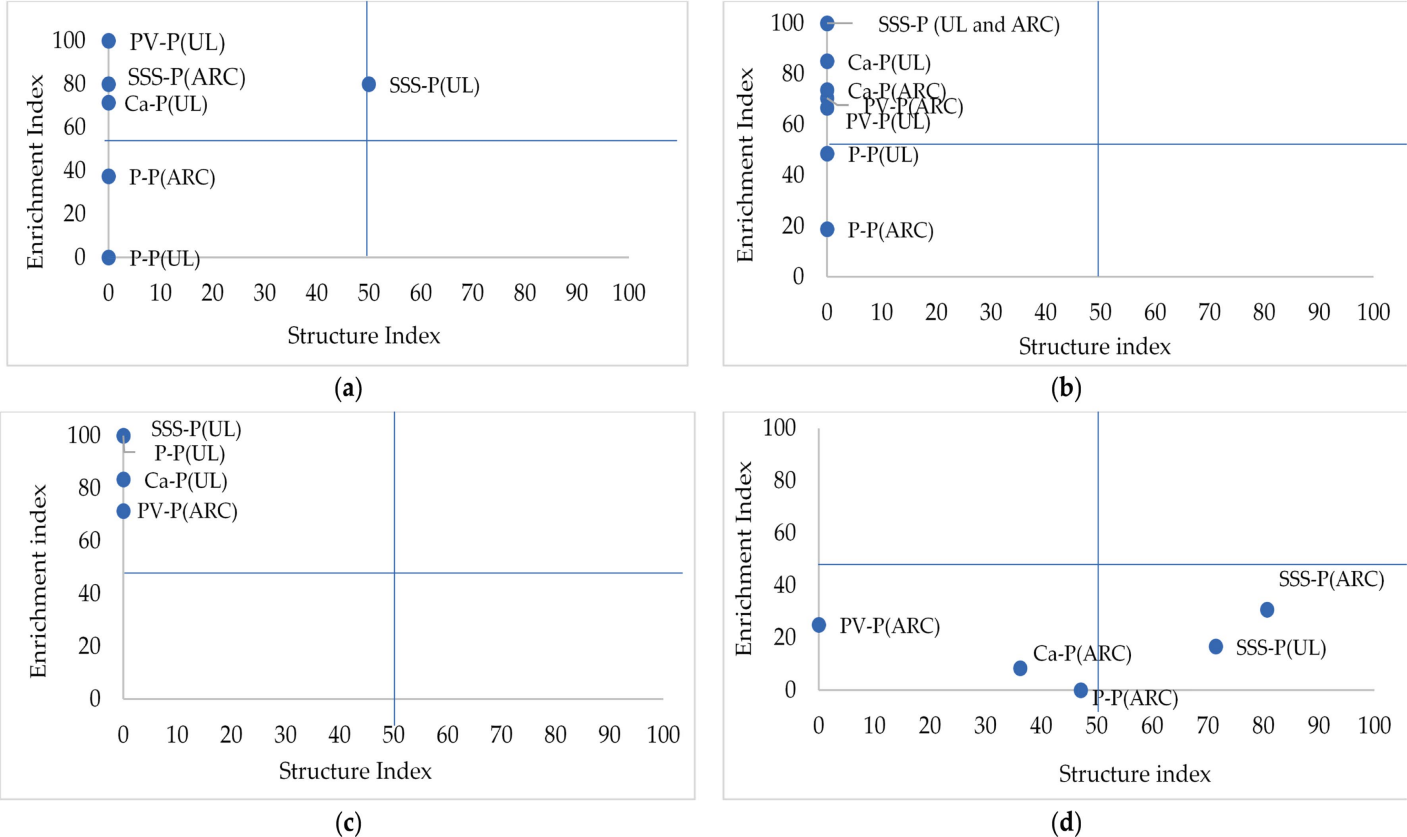
Nematode faunal profiles of soil samples collected from sequence 1 **(a)**, sequence 2 **(b)**, sequence 3 **(c)**, and sequence 4 **(d)** at the University of Limpopo (UL) and Agricultural Research Council (ARC) fields where P-P = potato monoculture, PV-P = potato-(Velum)-potato, Ca-P = *Cucumis africanus*-potato, and SSS-P = sweet stem sorghum-potato cropping sequences.

Soil samples from the different cropping sequences at UL and ARC-VIMP were grouped in quadrant A, C, or D (Figure 3). From Sequences 1 to 3, potato (Velum)-potato, sorghum-potato and *C. africanus*-potato plots were grouped in Quadrant A, whereas potato monoculture plots normally occurred in Quadrant D. However, in Sequence 4 potato (Velum)-potato, potato monoculture and *C. africanus*-potato were all grouped in Quadrant D, whereas sorghum-potato was found in Quadrant C (Figure 3). Soil samples not shown in the diagrams were not enriched or structured, hence they belonged to quadrant D.

## 4 Discussion

### 4.1 Effect of different cropping systems on final tuber yield

The effect of RKN on the growth and development of various crops has been studied extensively (Chiuta et al., 2025). The different cropping systems showed significant effects on tuber yield mass at both locations. Tuber yield from potato-(Velum)-potato and *C. africanus*-potato sequences were significantly higher than that in the potato monoculture and sorghum-potato sequence at UL and ARC. This shows that *C. africanus* can be used to manage RKN in potato-based cropping systems, as previously alluded to by Chiuta et al. (2025). High tuber yield recorded in potato-(Velum)-potato and *C. africanus*-potato sequences could be attributed to the effectiveness of these cropping systems in reducing the carry-over nematode population densities. In studies by Kimpinski et al. (2000) and Wang et al. (2012), the inclusion of post-infection nematode-resistant marigold (*Tagetes* species) cultivars in potato-based cropping systems significantly reduced nematode population densities and subsequently increased tuber yield by 14 and 23%, respectively. Similarly, McSorley et al. (1994) demonstrated that including different nematode-resistant crops in a cropping system significantly affected the targeted PPN population densities, subsequently influencing the overall yield of the successor crops.

Potato monoculture and sorghum-potato plots which recorded significantly higher RKN population densities, exhibited lower tuber yield mass. Therefore, low yield was attributed to nematode damage. Similarly, Crow et al. (2001) observed no tuber yield differences between potato monoculture and pre-infection resistant, sudan grass (*Sorghum drummondii*)-potato cropping sequence established on *M. incognita* infested fields. However, Lopez-Lima et al. (2013) observed no significant differences in potato tuber yield regardless of the efficacy of the treatment crop in managing the carry-over population densities of the target nematode. In such instances, other unquantified abiotic and biotic factors could have affected the final tuber yield mass in the test cropping systems. For example, poor soil structure, soil sickness due to accumulation of toxic allelochemicals released by the preceding crop, low soil fertility and other soil-borne diseases such as blight can reduce tuber yield in different cropping sequences (Koch et al., 2020).

### 4.2 Soil organic carbon, bacterial functional diversity and enzyme activity

Generally, poor soil health was observed under potato (Velum)-potato and potato monoculture as exhibited by low soil organic carbon, bacterial diversity and enzyme activity. On the contrary, soil health was promoted under *C. africanus*-potato and sorghum-potato cropping systems. Therefore, crop diversification in potato-based cropping system positively influenced soil health compared to potato monocropping. Indisputably, increasing crop diversity enriches the rhizosphere as different organic deposits accumulate subsequently enhancing bacterial diversity soil microbial diversity (Li et al., 2025; Liu et al., 2023).

The high carbon sources released into the rhizosphere can largely influence the microhabitats of the microorganisms by significantly changing the soil physicochemical properties (Yang et al., 2020). For example, *C. africanus* plants produce cucurbitacin B (C_32_H_46_O_8_), a carbon-rich tetracyclic triterpenoid which occurs in all organs of the plant (Shadung and Mashela, 2016). Although there are no known studies that ascertain the release of cucurbitacin B as a root exudate, plant residues (root and leaves) decomposition and release of root secretions could still be a great source of carbon deposit in the soil. Cucurbitacin B has been associated with the reduction of PPN (Mashela et al., 2017), but there is limited information on its overall effect on microbial diversity, particularly within the rhizosphere of actively growing *C. africanus* plants.

Similarly, most sorghum varieties secrete sorgoleone (C_22_H_30_O_4_), a carbon-rich root exudate throughout the entire growth cycle and it is readily mineralized by soil microbes existing in different soil types (Durand et al., 2018; Habig et al., 2018). Moreover, the deep and extensive root system of sorghum and *C. africanus* plants (Collange et al., 2011; Navarrete et al., 2016; Pofu, 2012) could provide adequate water and nutrients for microbial growth and diversity (Navarrete et al., 2016). Hence, the low microbial richness and evenness under potato monoculture with or without Velum application could be due to limited organic carbon sources.

Alternatively, potato monocropping causes the accumulation of toxic root secretions such as phthalic and palmitic acid, which promote microbial population imbalance (Qin et al., 2017). The lack of microbial diversity in the potato (Velum)-potato cropping system could be exacerbated by the application of synthetic chemical as previously reported in a similar study (Ashworth et al., 2017a). In this study, potato monoculture and potato (Velum)-potato were mapped in different quadrants of the PCA biplot at both UL and ARC locations. As such, the effects of these two cropping systems were significantly different, and this can be explained by the presence or lack of Velum Prime.

In other studies, monocropping of potato, maize (*Zea mays* L.), or soybean (*Glycine max* L.) did not compromise microbial diversity (Ashworth et al., 2017b; Miller et al., 2019). Thus, cropping sequence effect on soil health can be influenced by several factors, especially under field conditions. For instance, the results from the current study showed that *C. africanus*-potato was more effective in improving soil health at ARC-VIMP than at UL and vice versa for sorghum-potato. This could be due to differences in prevailing environmental conditions, edaphic factors, plant growth rate, field history, lack of soil homogeneity, initial microbial inoculum, *etc.* (Hennion et al., 2019; Yang et al., 2020). However, the use of Biolog Ecoplates offers inferences about culturable communities only, neglecting fastidious bacteria (Grządziel et al., 2018). As such, future research should validate these findings through modern molecular techniques.

The reduction in soil organic carbon and microbial diversity subsequently resulted in a decrease in ß-glucosidase, urease and acid phosphatase enzyme activity in potato monoculture and potato (Velum)-potato cropping sequences. Similarly, low ß-glucosidase, urease and acid phosphatase enzyme activities were reported under potato monoculture (Qin et al., 2017), and this was attributed to reduced food substrate diversity (Yu et al., 2022). In contrast, Malobane et al. (2020) observed no significant difference in enzyme activities under short-term crop rotation.

### 4.3 Nematode communities as an indicator for soil health

The MI of most cropping sequences at UL and ARC-VIMP were very low (≤ 2), indicating disturbed, enriched environments which are generally predominated by r-strategist nematodes (Ürkmez et al., 2014). Generally, environmental disturbances are high in most agricultural soils due to the various cultural practices that are implemented (Gupta et al., 2019). However, sorghum-potato cropping system recorded moderate MI values in Sequence 4, thereby demonstrating improved soil stability over time as evidenced earlier by increased soil organic carbon, microbial diversity and enzyme activity. These results corroborate with Eberly et al. (2024) report on sorghum-cotton (*Gossypium hirsutum* L.) crop sequence.

Soil samples with low MI are mostly associated with high PPI (DuPreez et al., 2022). In this study, PPI were approximately similar in all cropping systems. According to Li et al. (2016), the reduction in PPN by different cropping systems could take longer under field conditions. The low CI observed on most of the treatment plots at UL and ARC confirmed that the decomposition pathways were dominated by bacteria. Similarly, bacteria dominated food webs were observed in soils where crop rotation was practiced (Zhong et al., 2016). Conversely, extremely high CI values were recorded under potato monoculture or when sole potato cultivation was done, thereby showing the dominance of fungal-dominated decomposition pathways. This agrees with other studies that observed the preponderance of fungi under tobacco (*Nicotiana tabacum* L.) and cucumber (*Cucumis sativus* L.) continuous cropping (Ding et al., 2024; Jin et al., 2019).

The low (≤ 2) Shannon diversity index values observed on all the plots showed low nematode species richness. Based on this criterion, the inclusion of non-host crops showed no improvement in soil health. This could be due to the short duration of the study (Yang et al., 2020) or high soil disturbance due to intensive cultural practices implemented in each cropping system (Liu et al., 2024). In other studies, changes in nematode diversity were only recognized after 10 years of crop rotation (Zhong et al., 2016). Theoretically, fields where crop rotation is practiced should have high nematode diversity (Malherbe and Marais, 2015); however, that is not always the case in real-world scenarios.

The faunal profiles of soils collected from all cropping sequences except potato monocropping had moderate to high enrichment with no structure (Gupta et al., 2019). High EI normally occurs in soils that are predominated by Ba1 bacterivores (Liu et al., 2024). Most agricultural soils are usually mapped into Quadrant A, since high soil disturbance is commonly associated with high enrichment, low SI and degraded food web condition (Habig et al., 2018; Gupta et al., 2019). Monocultured plots were also unstable but highly resource-depleted, congruent with observations made on maize and wheat (*Triticum aestivum* L.) monocultures (DuPont et al., 2014; McDaniel and Grandy, 2016). The low enrichment could be due to reduced resource availability under continuous cropping as previously shown in this study. Additionally, potato monocropping created a conducive environment for high fungal activity, as observed in similar studies (Hu et al., 2019; Zhong et al., 2016), showing the dominance of fungal-dominated decomposition pathways.

The faunal profile exhibited in Sequence 4 had a different trend where all cropping systems were grouped in Quadrant D, except sorghum-potato in Quadrant C. Potato (Velum)-potato and potato monocropping plots showed the lowest stability and enrichment, respectively. At this juncture, the continuous application of Velum might have disturbed the presence of free-living nematodes, thereby changing community structure. These findings were consistent with observations made when Velum Prime was used to control nematodes in bermudagrass (*Cynodon dactylon* L.) (Waldo et al., 2019), and when other non-fumigant chemicals were applied (Grabau and Chen, 2016; Gupta et al., 2019; Zhong et al., 2016). However, Jansen (2014) observed no negative effect on free-living nematodes when synthetic chemicals were applied, thereby showing substantial variability in the impact spectrum of various nematicides.

Based on the results, sorghum-potato cropping sequences resulted in well-structured but resource-depleted soils (Ferris et al., 2011), considering that nematodes with high c-*p* values were observed over time. The extensive, fibrous root system and sorgoleone exudation from sorghum potentially increased food resources for microbial communities’ growth which subsequently become food source for high trophic level nematodes (Collange et al., 2011).

## 5 Conclusion

Nematode management strategies that improve soil health are desirable under sustainable agriculture. The different cropping systems showed a significant effect on final tuber yield mass at both locations, with potato (Velum)-potato and *C. africanus*-potato recording significantly higher tuber yield than potato monoculture and sorghum-potato. Soil from all plots was highly disturbed and dominated by bacterial decomposition pathways. However, crop diversification increased soil organic carbon content, microbial diversity and enzyme activity. Overall, sorghum-potato effectively improved soil health and structure over time, better than the other cropping systems. The study hinted possible shifts in nematode community structure associated with Velum application. However, long-term research is recommended to clarify this conjecture. Additionally, due to the complexity of plant-microbial interaction studies, continuous measurement of climatic and other edaphic factors that influence microbial activities should be done to better understand the influence of these cropping systems on overall soil health. Furthermore, the use of advanced molecular techniques can accurately determine and validate the microbial communities occurring in each cropping system, given the limitations associated with the conventional methods used in this study.

## Data Availability

The raw data supporting the conclusions of this article will be made available by the authors, without undue reservation.
