# Supplementary material for: Short-term crop rotation interactive effects on soil microbial communities and potato yield under field conditions
Source: Front Microbiol. 2026 Apr 29;17:1807765. doi: 10.3389/fmicb.2026.1807765 (PMC13167708; doi:10.3389/fmicb.2026.1807765)
Supplement: Supplementary file 1 [file Table_1.docx]

Supplementary tables

Table S1: Eigenvalues for University of Limpopo (UL) and Agricultural Research Council-Vegetable, Industrial and Medicinal Plants (ARC-VIMP) biplots.

| Location | UL | | | ARC-VIMP | | |
| --- | --- | --- | --- | --- | --- | --- |
|  | PC1 | PC2 | PC3 | PC1 | PC2 | PC3 |
| Eigenvalue | 4.655 | 1.117 | 0.229 | 3.247 | 2.126 | 0.626 |
| Variability (%) | 77.576 | 18.615 | 3.809 | 54.119 | 35.441 | 10.439 |
| Cumulative % | 77.576 | 96.191 | 100.000 | 54.119 | 89.561 | 100.000 |

Table S2: Factor loadings for all measured active variables at University of Limpopo (UL) and Agricultural Research Council-Vegetable, Industrial and Medicinal Plants (ARC-VIMP)

|  | Location | | | | | |
| --- | --- | --- | --- | --- | --- | --- |
|  | UL | | | ARC-VIMP | | |
| Active variables | F1 | F2 | F3 | F1 | F2 | F3 |
| ß-glucosidase | **0.883** | 0.469 | 0.028 | 0.153 | **0.955** | -0.255 |
| Acid Phosphatase | **0.959** | -0.263 | -0.107 | **0.924** | -0.370 | 0.091 |
| Urease | 0.693 | **0.702** | -0.165 | 0.168 | **0.942** | 0.290 |
| Shannon (H') | **0.802** | -0.528 | -0.279 | **0.927** | 0.356 | -0.120 |
| Evenness (E) | **0.991** | -0.007 | 0.137 | **0.854** | -0.221 | -0.470 |
| Soil Organic Carbon | **0.923** | -0.237 | 0.304 | **0.867** | -0.119 | 0.484 |

Where F: Factor loading, values in bold correspond for each observation to which the factor loading is the largest.
